# Pathology and tissue tropism of natural West Nile virus infection in birds: a review

**DOI:** 10.1186/1297-9716-44-39

**Published:** 2013-06-03

**Authors:** Virginia Gamino, Ursula Höfle

**Affiliations:** 1 SaBio Instituto de Investigación en Recursos Cinegéticos IREC, (CSIC-UCLM-JCCM) Ronda de Toledo s/n, Ciudad Real 13005, Spain

## Abstract

West Nile virus (WNV) is a globally distributed arthropod-borne flavivirus capable of infecting a wide variety of vertebrates, with birds as its natural reservoir. Although it had been considered a pathogen of little importance for birds, from the 1990’s, and especially after its introduction in the North American continent in 1999, thousands of birds have succumbed to West Nile infection. This review summarizes the pathogenesis and pathology of WNV infection in birds highlighting differences in lesion and antigen distribution and severity among bird orders and families. Despite significant species differences in susceptibility to infection, WNV associated lesions and viral antigen are present in the majority of organs of infected birds. The non-progressive, acute or more prolonged course of the disease accounts for part of the differences in lesion and viral antigen distribution and lesion severity. Most likely a combination of host variables and environmental factors in addition to the intrinsic virulence and pathogenicity of the infecting WNV strain influence the pathogenesis of the infection.

## Table of contents

1. Introduction

2. Etiology

3. Eco-epidemiology

4. Pathogenesis in birds

5. Pathology of natural WNV infection in birds

5.1. Central nervous system

5.2. Eye

5.3. Peripheral nervous system

5.4. Heart

5.5. Spleen and other lymphoid organs

5.6. Liver

5.7. Kidney

5.8. Lung

5.9. Gastrointestinal tract

5.10. Endocrine system

5.11. Gonads

5.12. Skeletal muscle

5.13. Skin

5.14. Bone marrow

6. Discussion and conclusion

7. Competing interests

8. Authors’ contributions

9. Authors’ information

10. Acknowledgements

11. References

## 1. Introduction

West Nile virus (WNV) is an arthropod-borne virus of the genus *Flavivirus* capable of infecting a wide variety of vertebrates, with birds as its natural reservoir [1]. It was first isolated in Uganda in 1937 from a woman with a febrile process [2]. During the 1960’s WNV was one of the most widely distributed flaviviruses in humans, birds and mosquitoes in Africa, the Middle East and south-western Europe, where it was considered a pathogen of little importance, causing subclinical infection or sporadic self-limiting outbreaks in horses and humans [3,4]. From the 1990’s the frequency and severity of human infections as well as the number of cases in other vertebrates including companion, farm and wild animals has increased [5]. In Europe, WNV causes disease in horses and humans, and recently sporadic mortality events have been reported primarily in birds of prey [6-8]. In contrast, after its introduction into North America in 1999, where it caused one of the most important outbreaks in New York, thousands of birds, horses and humans have died of the disease [9,10]. Nowadays it is one of the most widely distributed flaviviruses in the world and an important public and animal health and conservation concern.

## 2. Etiology

West Nile virus belongs to the genus *Flavivirus* of the family *Flaviviridae.* It has been serologically classified within the Japanese encephalitis antigenic group. The viral positive sense single-stranded RNA genome encodes a polyprotein which is translated into different structural (envelope protein E, membrane precursor protein prM and capsid protein C) and non-structural (NS1, NS2a, NS2b, NS3, NS4a, NS4b, and NS5) proteins that play an important role in the viral host range, tissue tropism, viral replication and assembly, and host immune system stimulation [11-13]. Based on phylogenetic analysis, WNV strains have been grouped into seven genetic lineages [14] but there are two major lineages, lineage 1 and lineage 2. Lineage 1 is widespread and contains isolates from Europe, America, the Middle East, India, Africa and Australia [15,16]. Lineage 2 had originally only been isolated in Southern Africa and Madagascar but has recently also been detected in Europe [6,17-19]. In general lineage 1 viruses were considered to be more virulent than lineage 2 viruses, however it has been demonstrated that both lineages contain neuroinvasive phenotypes and some of the recent outbreaks in Europe, that also affect birds, have been caused by the latter [6,18,20] (Table 1). Increased virulence of WNV strains has been associated to genetic and aminoacidic changes in structural and non-structural proteins. Substitution to a proline in position 249 of the non-structural protein NS3 has been associated with increased virulence in WNV lineage 1 strains in American crows (*Corvus brachyrhynchos*) and the same substitution has been detected in recent lineage 2 strains causing important outbreaks in Europe [15,21-24].

**Table 1:** Bird species found dead or with symptoms of encephalitis in Europe in which the presence of WNV has been evidenced by real time RT-PCR or by virus isolation

**Year**	**Country**	**Order**	**Family**	**Species**	**Lineage**	**Ref**
2001-2004	Spain	Falconiformes	*Accipitridae*	Spanish imperial eagle	1	[7]
				(*Aquila adalberti*)		
2003	Hungary	Anseriformes	*Anatidae*	Domestic goose	1	[6,25]
				(*Anser anser domesticus*)		
2004	Hungary	Falconiformes	*Accipitridae*	Goshawk	2	[6,26]
				(*Accipiter gentilis*)		
	France	Passeriformes	*Corvidae*	Common magpie	1	[27]
				(*Pica pica*)		
			*Passeridae*	House sparrow		
				(*Passer domesticus*)		
2005	Hungary	Falconiformes	*Accipitridae*	Goshawk	2	[20,28]
				Sparrow hawk		
				(*Accipiter nisus*)		
2007	Spain	Falconiformes	*Accipitridae*	Golden eagle	1	[28]
				(*Aquila chrysaetos*)		
				Bonelli’s eagle		
				(*Aquila fasciata*)		
2008	Austria	Falconiformes	*Accipitridae*	Sparrow hawk	2	[20,28]
				(*Accipiter nisus*)		
				Goshawk		
				(*Accipiter gentilis*)		
			*Falconidae*	Gyrfalcon		
				(*Falco rusticolus*)		
		Psittaciformes	*Strigopidae*	Kea		[28]
				(*Nestor notabilis*)		
	Italy	Charadriiformes	*Laridae*	Herring gull	1	[29]
				(*Larus argentatus*)		
		Columbiformes	*Columbidae*	Pigeon		
				(*Columba livia*)		
		Passeriformes	*Corvidae*	Common magpie		
				(*Pica pica*)		
				Carrion crow		
				(*Corvus corone*)		
				Eurasian jay		
				(*Garrulus glandarius*)		
		Suliformes	*Phalacrocoracidae*	Great cormorant		
				(*Phalacrocorax carbo*)		
2009	Austria	Falconiformes	*Accipitridae*	Goshawk	2	[20]
				(*Accipiter gentilis*)		
2011	Italy	Columbiformes	*Columbidae*	Collared dove	2	[18]
				(*Streptopelia decaocto*)		
2012	Italy	Falconiformes	*Accipitridae*	Goshawk	2	[30]
				(*Accipiter gentilis*)		

## 3. Eco-epidemiology

The virus is maintained in an enzootic ornitophilic-mosquito-bird cycle [31]. Migratory birds are considered to play an important role in the local and long distance viral dispersal [32-35]. The most susceptible species to infection belong to the family *Corvidae* of the order Passeriformes such as the American crow, the blue jay (*Cyanocitta cristata*), the black-billed magpie (*Pica hudsonia*) or the fish crow (*Corvus ossifragus*) [9,36]. Some species of this order such as the American robin (*Turdus migratorius*) or the house sparrow (*Passer domesticus*) are also considered the main reservoir of WNV in urban areas of North America and Europe [37,38]. Other vertebrates susceptible to infection include amphibians, reptiles and mammals, among which the virus can be especially pathogenic for equids and humans. However, most of them are considered accidental or dead-end hosts as they rarely develop sufficient viremia to infect feeding mosquitoes [5,39]. Although WNV transmission in birds occurs primarily by mosquitoes of the genus *Culex*, transmission through infected prey or contaminated water consumption and horizontal contact transmission have also been documented [36,40-43]. The virus is maintained in a silent mosquito-bird cycle in natural habitats (“rural cycle”) but can cause outbreaks in humans and horses when it is introduced in humanized habitats where mosquitoes with a wider host range are present, establishing an “urban cycle” [44]. In endemic areas wild bird infection usually starts in spring and early summer, mortality peaks from midsummer to early fall, and human and horse cases take place a few weeks after the onset of bird mortalities [45].

Before the 1990’s WNV had been isolated in different parts of Europe, Asia and Africa but was rarely associated with clinical disease. Although some studies conducted in Africa in the 1950’s underlined the role of birds as amplifying hosts and mortality was reported in host competence studies, especially in hooded crows (*Corvus corone sardonius*) [46], prior to 1997 WNV was considered low-pathogenic for birds [47]. In 1998 a large outbreak took place in Israel with high mortality in birds, particularly in geese (*Anser anser domesticus*), and WNV was isolated from the brain of dead free-living storks (*Ciconia ciconia*) [48]. Currently, WNV is considered endemic in Europe [44]. Although viral circulation has been reported in many bird species, cases of encephalitis or mortality due to viral infection in wild birds have been sporadic [47] and observed mainly in raptors (Table 1). In North America WNV was detected for the first time in 1999 in New York [49] and since then it has caused thousands of bird deaths being detected in at least 326 bird species [50] (Table 2). WNV became endemic within ten years of its introduction into this continent [51]. The epidemiological behavior in North America, with an intense viral circulation, differs from the epidemiological behavior in other parts of the world including Europe, and the reasons remain largely unknown [44]. Viral phenotype, host heterogeneity in the area, vector abundance, feeding activity or host preference, and host susceptibility have been identified as possible modulating factors [11,44,52-55]. Host susceptibility to infection has been associated to geographic range, mating and breeding systems, body size, migratory behavior and to co-evolution with the virus or antigenically-related flaviviruses [56-60].

**Table 2:** Bird orders and families from North America in which mortality due to WNV infection has been demonstrated

**ORDER**	**FAMILY**
**Anseriformes**	*Anatidae*
**Apodiformes**	*Apodidae, Trochilidae*
**Caprimulgiformes**	*Caprimulgidae*
**Casuariiformes**	*Dromaiidae*
**Charadriiformes**	*Charadriidae, Laridae*
**Ciconiiformes**	*Ardeidae, Cathartidae, Ciconiidae, Threskiornithidae*
**Columbiformes**	*Columbidae*
**Coraciiformes**	*Alcedinidae, Bucerotidae*
**Cuculiformes**	*Cuculidae*
**Falconiformes**	*Accipitridae, Falconidae*
**Galliformes**	*Numididae, Odontophoridae, Phasianidae*
**Gaviiformes**	*Gaviidae*
**Gruiformes**	*Aramidae, Gruidae, Rallidae*
**Musophagiformes**	*Musophagidae*
**Passeriformes**	*Bombycillidae, Cardinalidae, Cinclidae, Corvidae, Emberizidae, Estrildidae, Fringillidae, Icteridae, Laniidae, Mimidae, Paridae, Parulidae, Passeridae, Sittadae, Sturnidae, Thraupidae, Troglodytidae, Turdidae, Vireonidae*
**Pelecaniformes**	*Pelecanidae, Phalacrocoracidae*
**Phoenicopteriformes**	*Phoenicopteridae*
**Piciformes**	*Picidae*
**Podicipediformes**	*Podicipedidae*
**Psittaciformes**	*Aratingidae, Cacatuidae, Psittacidae*
**Sphenisciformes**	*Spheniscidae*
**Strigiformes**	*Strigidae, Tytonidae*
**Struthioniformes**	*Struthionidae*
**Tinamiformes**	*Tinamidae*

## 4. Pathogenesis in birds

Most of the information about the pathogenesis of WNV infection is derived from experimental studies done in mammals, mainly rodents. In these hosts, after mosquito blood-feeding, WNV replicates in the skin and is transported by Langerhans dendritic cells to draining lymph nodes. There the virus replicates and primary viremia and peripheral organ dissemination take place [61]. On the contrary, the exact mechanism and sites of WNV replication in avian hosts are still not well understood. It is supposed that, as in mammals, it replicates locally at the inoculation site and is rapidly distributed to all organ systems [62]. However, it has been demonstrated that the virus can be detected in the blood as early as 30–45 min after the mosquito feeding period, suggesting that in birds local replication is not necessary for the primary viremia [63]. In general, WNV can be isolated from the blood of infected birds at one day post-infection (dpi) (one day later in less susceptible species such as chicken or turkeys or in the case of oral infection) [34,64,65]. Viremia can peak as early as 2–3 dpi in geese and some Passeriformes such as crows and jays, or 4–6 dpi in raptors, owls and chicken [43,64-67]. Mean peak viremia is higher in those birds that finally succumb to the disease [38]. WNV can be detected in the blood until day 6–7 pi in geese, passerines and owls and up to 10 dpi in raptors and turkeys [40,66-69]. Little is known about innate avian host defenses against WNV infection. In mammals, this includes IFN production, complement activation, phagocytosis and cytotoxicity, with the participation of macrophages, neutrophils, NK cells and γδ T cells [61,70]. WNV infects all major organ systems and a wide variety of individual cell types. The targeting of cells of the mononuclear phagocytic system may play an important role in the pathogenesis in infected birds, as the virus can replicate within these cells and disseminate to a wide variety of tissues [64,71]. As early as 1 dpi WNV can be detected in the spleen of crows and one day later it is widely distributed. In this species the highest viral titer in tissues can be reached on day 4 pi, decreasing from day 5 pi [64]. On the contrary in falcons and owls maximum viral titer in tissues can be delayed until day 7–8 pi, decreasing from day 9 pi, and while in falcons the virus remains detectable on day 14 pi, it can be totally cleared from most tissues of owls [42,66,72].

Flaviviruses have evolved different strategies that modulate the host immune response [73] and depending on the effectiveness of this response and, therefore, the level of viremia, WNV can reach the central nervous system (CNS). While WNV can be detected in the brain of crows as early as 2 dpi, in owls it may not be present before 5 dpi [64,66]. In the former maximum viral titer in this tissue can be reached on day 4 pi while this may not take place in the latter until day 8 pi or until 14 dpi in falcons [64,66,72]. The mechanisms by which WNV crosses the blood–brain barrier and reaches the CNS remain unknown. Some authors have suggested that TNF-α mediated change in endothelial cell permeability may facilitate its entry, at least in mammals [74]. Other mechanisms proposed are the infection of or passive transport through the endothelium or epithelial cells of the choroid plexus [75], direct axonal retrograde transport from olfactory or motor neurons [76,77] or transport by infected immune cells [78]. In birds, WNV detection in endothelial cells by immunohistochemistry (IHC) and the presence of perivascular inflammatory infiltrates [79,80] may indicate that WNV reaches the CNS via the blood stream and infects endothelial cells, although viral transport through infected immune cells may also be probable [64].

The development of clinical disease is caused by the invasion of the CNS and/or other major organs such as the liver, spleen, kidney and heart [71]. In most cases, clinical signs appear approximately on 5 dpi in experimentally WNV infected birds, but may be absent both in experimental and natural infections [36,42,81-84]. Unspecific clinical signs include depression, anorexia, dehydration and ruffled feathers. In more than 60% of infections convulsions are present, approximately in 30% appear ataxia, abnormal head posture and head movements, and in up to 20% tremors, uncoordinated flight, paresis and disorientation are observed [71,82,85-89]. The development of impaired vision and blindness is common in raptors and owls [89-91]. Long-term sequelaes have been detected in long-lived birds such as raptors, in which relapses of neurologic signs, feather pulp abnormalities and abnormal molt can persist up to 4 years [92], and may have a negative impact on the longevity of these species [42,92].

Systemic infection of the host also activates the adaptive immune response that includes B and T cell activation and antibody production [61,70]. In most bird species seroconversion can first be detected between 4–6 dpi [64,84] and neutralizing antibodies, at least in some species, persist for longer than a year and can be transferred from adult females to their progeny [93-96]. In mice it has been indicated that CD8^+^ and CD4^+^ T lymphocytes combined with antibodies and chemokines are essential for viral clearance from the CNS and peripheral organs [97]. In birds it has been demonstrated that the virus can persist in different organs such as the spleen, kidney, eye, brain or the skin [36,98-101]. Wheeler et al. [98] demonstrated that viral persistence in naturally infected Passeriformes can last for 4 months extending to up to 6 months in experimentally infected birds. Viral persistence consequences for the avian host are still not clear [98] but it can play an important role in viral overwintering and mosquito infection in case of host immune defenses impairment and viremia recrudescence [101-103].

In case of host defense failure, death often occurs within 24 h or two to four days after the onset of clinical signs in experimentally or naturally infected birds, respectively [25,26,36,83]. Sometimes death is not directly related to WNV associated tissue lesions but to concurrent disease such as trauma or bacterial, fungal or parasitic infections [7,42,88,92].

The outcome of WNV infection and development of lesions and disease in individual birds depend mainly on viral and host factors. Glycosylation in the E protein of WNV has been demonstrated to increase peripheral viremia and virulence for birds and neuroinvasiveness in rodents [104,105]. Brault et al. [21] experimentally demonstrated that a T_249_P NS3 substitution in the WNV NY99 strain increased virulence for American crows. However, this substitution appears not to have the same effect for some other North American bird species and an experimental study has shown that this substitution alone in European WNV strains does not necessarily lead to an increased virulence for susceptible European birds [84]. Papa et al. [23] related the H_249_P NS3 substitution to the increased virulence of the lineage 2 WNV strain that caused the outbreak in humans in Greece in 2010 [106], but the role of this substitution in the virulence for birds is not clear, as lineage 2 strains that have caused mortalities in birds do not show this change [18,23,30]. Therefore, the presence of other genetic markers of virulence could be implicated [22,107].

The existence of natural infections of goshawks (*Accipiter gentilis*) by either WNV lineage 1 and 2 strains allows comparison of the associated pathology. In both cases viral cellular and tissue tropism is similar but lesion localization and nature slightly differ. Thus for example splenic lymphoid depletion, nephritis and hepatitis present in lineage 2 infected goshawks are not described in those infected by lineage 1 which in change have hepatic and myocardial necrosis that are not described in those infected by lineage 2 [20,26,91].

Another determinant factor is the ability of the WNV strain to replicate at the high corporal temperature of the avian host, as has been demonstrated for the WNV NY99 genotype [108]. Moreover, viral inoculation dose should not be forgotten, although dose-dependent pathological differences are not always evidenced in experimental infections [67]. Finally, infection route can influence pathogenicity of WNV infections, as some authors have indicated that in orally infected birds tissue lesions are less severe and the incidence of clinical signs or mortality is lower than in those subcutaneously infected [36,42]. Bird species is an important host factor that influences the pathogenesis of WNV infection, as has been demonstrated by numerous authors [36,71,79]. Also, the immune status of the host plays an essential role in the pathogenesis, as it determines the capacity of the host to clear the virus [61]. It can be modulated by the presence of previous immunity against the virus or cross immunity against antigenically-related flaviviruses, the presence of concurrent diseases, the influence of hormonal factors and stress, as well as age [56,83,109-112]. Age not only determines the maturity of the immune system but also the expression of cellular receptors important for viral entry and intracellular factors that participate in the pathogenesis of the disease [113]. Finally, it has been demonstrated in avian and rodent models that genetic factors influence host susceptibility to flaviviral infections [114,115].

## 5. Pathology of natural WNV infection in birds

The pathology of natural WNV infection in birds has been documented in more than 57 species of at least 13 families and 10 orders.

Because of the wide cellular and tissue tropism of WNV, there are no pathognomonic macroscopic lesions. Bird species with a low susceptibility to WNV infection such as chicken may have no observable macroscopic lesions at necropsy [65], and those highly susceptible such as crows or jays will die rapidly after a short incubation period and may have few acute or no observable lesions [80,81]. In contrast, birds that survive longer will have more pronounced macroscopic lesions, that may be chronic in the case of individuals that survive days to months [79,91]. Emaciation, dehydration, multiorgan hemorrhages, petechiae and congestion are the most characteristic macroscopic changes, but also splenomegaly, hepatomegaly, myocardial pallor and pale mottling in the liver, spleen or kidney. Cerebral atrophy and malacia can be observed in raptors [42,71,91].

**Figure 1:**
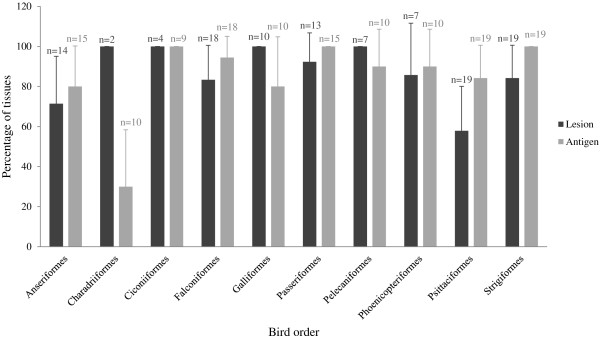
**Lesion extension and antigen distribution among examined tissues in WNV infected bird orders as reported in the reviewed literature.** Each column represents the percentage of tissues collected (n) in which lesions or antigen is present.

In the vast majority of bird families, microscopic lesions due to WNV infection are predominantly found in the CNS, heart, kidney, spleen and liver (Table 3). Pathological changes can be the result of the direct effect of the virus or secondary to the host inflammatory response [113,116,117]. Lymphoplasmacytic and histiocytic infiltrates, cellular degeneration and necrosis, and hemorrhages are the main microscopic findings. However, there are differences in antigen and microscopic lesion severity and distribution pattern depending on the species infected or the dpi the analyzed individual died [1] (Table 3 and Figure 1). Highly susceptible species such as crows and jays usually have large amounts of virus in the blood and widely distributed in major organs. Microscopic changes in these cases are acute with minimal inflammatory reaction and can be absent in the CNS [64,80,118]. In contrast, birds that survive longer such as raptors and owls may develop chronic lesions that can be also found in the CNS [42,91].

**Table 3:** Distribution of specific lesions in tissues of naturally WNV infected birds

Order*	**ANSE**	**CHAR**	**CICO**	**FALC**		**GALL**	**PASS**			**PELE**	**PHOE**	**PSIT**	**STRI**
**Family♦**	**ANAT**	**LARI**	**ARDE**	**ACCI**	**FALC**	**PHAS**	**CORV**	**LANI**	**PASS**	**PHAL**	**PHOE**	**PSIT**	**STRI**
**Reference**	[25,71,124]	[71]	[71]	[20,26,42,71,89,91,117,119]	[20,42]	[71,83,122]	[71,80,118,120]	[81]	[121]	[71]	[71]	[87,123]	[42,66,71,79,82,90,91,119]
**TISSUE/Lesion**													
**BRAIN**													
Neuronal necrosis-degeneration	+	-	ND	+	-	-	-	-	-	-	-	-	+
Neuronal vacuolization	-	+	ND	+	-	+	+	-	-	+	-	-	-
Meningeal inflammatory infiltrates	+	-	ND	+	-	-	+	-	-	+	-	-	+
Gliosis	+	+	ND	+	+	+	+	+	-	-	+	+	+
Perivascular cuffs	+	+	ND	+	-	+	+	+	+	-	+	+	+
Glial nodules	+	+	ND	+	-	+	+	+	-	-	+	-	+
Vasculitis	-	-	ND	+	-	-	+	-	+	-	-	-	-
Hemorrhage	-	-	ND	-	-	+	-	-	-	+	-	-	+
**SPINAL CORD**													
Neuronal necrosis-degeneration	+	ND	ND	-	ND	ND	ND	NT	ND	ND	ND	ND	+
Inflammatory infiltrates	+	ND	ND	+	ND	ND	ND	NT	ND	ND	ND	ND	+
**EYE**													
Retinal cell necrosis	NT	NT	NT	+	NT	NT	ND	NT	NT	NT	NT	-	-
Inflammatory infiltrates	NT	NT	NT	+	NT	NT	ND	NT	NT	NT	NT	+	+
**PERIPHERAL NERVOUS SYSTEM**													
Inflammatory infiltrates	ND	ND	ND	+	NT	ND	-	NT	NT	ND	ND	ND	+
**HEART**													
Myofibril necrosis	+	-	-	+	-	+	+	-	-	-	-	+	+
Myocytolisis-mineralization	+	+	+	+	-	+	+	-	-	+	+	+	-
Inflammatory infiltrates	+	+	+	+	+	+	+	+	-	+	+	+	+
Vasculitis	-	-	-	-	+	-	+	-	-	-	-	-	-
Hemorrhage	-	+	+	+	-	+	+	-	-	+	+	+	-
**SPLEEN**													
Lymphoid cell necrosis/apoptosis	+	ND	+	+	-	+	+	+	-	+	ND	+	+
Fibrin deposition	-	ND	+	-	-	+	-	+	-	+	ND	-	+
Lymphoid cell depletion	-	ND	-	+	-	-	-	-	-	-	ND	-	+
Hemosiderosis	+	ND	-	+	-	+	+	-	+	-	ND	-	+
Hemorrhage	+	ND	+	-	-	-	-	-	-	+	ND	-	-
**LIVER**													
Hepatocyte necrosis	-	ND	ND	+	-	-	+	+	+	+	+	+	+
Kupffer cell necrosis	-	ND	ND	-	-	-	+	-	-	-	-	-	-
Inflammatory infiltrates	-	ND	ND	+	+	+	+	+	-	-	-	+	+
Vasculitis	-	ND	ND	-	+	-	-	-	-	-	-	-	-
**LIVER**													
Biliary duct hyperplasia	-	ND	ND	+	-	-	-	-	-	-	+	-	-
Hemosiderosis	-	ND	ND	+	-	+	+	-	+	-	-	-	+
Hemorrhage	-	ND	ND	+	-	-	+	-	-	-	-	-	-
**KIDNEY**													
Tubular epithelial cell necrosis	-	ND	-	+	-	-	+	ND	+	-	-	+	+
Glomerular cell necrosis	-	ND	-	-	-	-	-	ND	-	-	-	+	+
Inflammatory infiltrates	+	ND	+	+	+	+	+	ND	+	+	+	+	+
Vasculitis	-	ND	-	-	+	-	-	ND	-	-	-	-	-
Hemorrhage	+	ND	-	-	-	-	-	ND	-	-	-	-	-
**LUNG**													
Cellular necrosis	-	ND	ND	+	-	-	+	-	-	ND	ND	-	+
Inflammatory infiltrates	-	ND	ND	+	-	+	+	-	+	ND	ND	-	+
Vasculitis	-	ND	ND	-	-	-	+	-	-	ND	ND	-	-
Edema	-	ND	ND	+	+	-	-	+	+	ND	ND	-	-
**GASTROINTESTINAL TRACT**													
Enterocyte necrosis	-	ND	ND	-	-	-	+	-	ND	-	-	-	+
Intestinal crypt cell necrosis	-	ND	ND	-	-	-	+	+	ND	+	+	+	+
Inflammatory infiltrates	-	ND	ND	+	-	+	+	-	ND	-	-	+	+
Hemorrhage	+	ND	ND	-	-	-	+	-	ND	-	+	-	-
**ENDOCRINE SYSTEM**													
Pancreatic acinar cell necrosis	+	ND	ND	-	ND	+	-	+	ND	-	+	+	+
Pancreatic inflammatory infiltrates	-	ND	ND	+	ND	+	-	-	ND	+	+	+	+
Adrenal gland cell necrosis	ND	ND	ND	-	NT	ND	-	NT	NT	ND	ND	+	-
Adrenal inflammatory infiltrates	ND	ND	ND	+	NT	ND	+	NT	NT	ND	ND	+	+
Thyroid gland cell necrosis	+	NT	NT	-	NT	NT	NT	NT	NT	NT	NT	ND	ND
Thyroid inflammatory infiltrates	-	NT	NT	+	NT	NT	NT	NT	NT	NT	NT	ND	ND
**OTHER LYMPHOID ORGANS**													
Bursal epithelial cell atrophy-apoptosis	-	NT	NT	+	NT	NT	NT	NT	-	NT	NT	ND	+
Bursal lymphoid cell atrophy-apoptosis	-	NT	NT	+	NT	NT	NT	NT	+	NT	NT	ND	+
Thymic lymphoid cell necrosis	+	NT	NT	NT	NT	NT	NT	NT	NT	NT	NT	-	+
**GONADS**													
Cellular necrosis	ND	ND	ND	-	-	ND	NT	ND	NT	ND	ND	-	+
Inflammatory infiltrates	ND	ND	ND	-	-	ND	NT	ND	NT	ND	ND	-	+
**SKELETAL MUSCLE**													
Myofibril degeneration-necrosis	ND	NT	NT	+	-	-	ND	NT	NT	NT	NT	+	+
Inflammatory infiltrates	ND	NT	NT	+	+	+	ND	NT	NT	NT	NT	+	+
Fibrosis	ND	NT	NT	+	-	-	ND	NT	NT	NT	NT	-	+
**SKIN**													
Inflammatory infiltrates	NT	NT	NT	ND	NT	NT	NT	ND	NT	NT	NT	+	-
**BONE MARROW**													
Cellular necrosis	NT	NT	NT	ND	NT	-	+	NT	NT	NT	NT	ND	ND
Hypercellularity	NT	NT	NT	ND	NT	+	-	NT	NT	NT	NT	ND	ND

*ANSE Anseriformes, *CHAR* Charadriiformes, *CICO* Ciconiiformes, *FALC* Falconiformes, *GALL* Galliformes, *PASS* Passeriformes, *PELE* Pelecaniformes, *PHOE* Phoenicopteriformes, *PSIT* Psittaciformes, *STRI* Sitrigiformes.

♦*ANAT* Anatidae, *LARI* Laridae, *ARDE* Ardeidae, *ACCI* Accipitridae, *FALC* Falconidae, *PHAS* Phasianidae, *CORV* Corvidae, *LANI* Laniidae, *PASS* Passeridae, *PHAL* Phalacrocoracidae, *PHOE* Phoenicopteridae, PSIT Psittacidae, STRI Strigidae.

ND: No described lesion. Tissues that were taken during necropsy but for which there are no lesions described.

NT: Tissue not tested. Tissue not analyzed in the necropsy.

+ Lesion present. Lesion described by at least one author for the tissue.

- Lesion absent. Lesion stated as absent or not specifically described by any author for the tissue.

### 5.1. Central nervous system

CNS lesions can appear on 8–9 dpi in falcons and owls, increasing their severity during the course of infection, and can be maintained chronically [42,66,72]. In the chicken, the appearance of lesions can be delayed until 21 dpi [65]. Lymphoplasmacytic and histiocytic meningoencephalitis characterized by gliosis, perivascular cuffing and glial nodules is the main microscopic finding. This inflammation is usually present in the molecular layer of the cerebellum and in some cases also in the cerebrum, mesencephalon and medulla oblongata. Neuronal necrosis and degeneration are usually found in raptors [42] and owls [119]. Vasculitis is detected in red-tailed hawks (*Buteo jamaicensis*), yellow-billed magpies (*Pica nuttalli*) and house sparrows [42,120,121] and hemorrhages are observed in guanay cormorants (*Phalacrocorax bougainvillea*), turkeys and owls [71,90,122] (Table 3). WNV antigen is usually detected by IHC in the CNS. Cells showing antigen labeling include neurons (including Purkinje cells) and glial cells as well as other inflammatory cells and vascular endothelial cells.

### 5.2. Eye

The eye is not routinely microscopically studied due to the difficulty of obtaining sections suitable for interpretation. However, it has been more closely examined in raptors and owls, probably because of the importance of vision in these species. Moderate to marked lymphoplasmacytic and histiocytic inflammatory infiltrates, disarray of the retinal pigmented epithelial cell layer and retinal cell necrosis and mineralization have been described in naturally WNV infected hawks [117]. Birds of the order Psittaciformes and Strigiformes only show inflammatory infiltrates that are mainly detected in the conjunctiva, iris, choroid, pecten and retina [90,123] (Table 3). WNV antigen is detected in different cells of the retina and in inflammatory infiltrating cells. Although Wünschmann et al. [80] described the presence of WNV antigen in endothelial cells of the pecten and choroid in the eye of infected American crows, they did not describe associated lesions in this species. In contrast, WNV antigen presence has not been documented in the eye of Psittaciformes [123].

### 5.3. Peripheral nervous system

WNV related lesions in the peripheral nervous system have only been described in raptors and owls [119]. Mononuclear or mixed inflammation is usually present in the sciatic nerve and in the myenteric, proventricular and ventricular ganglia (Table 3). Neuronal positivity can be detected by IHC.

### 5.4. Heart

Myocardial lesions may not be detected in falcons until 14 dpi, but appear earlier in owls (8 dpi) and crows (6 dpi) [64,66,72]. Lymphoplasmacytic and histiocytic myocarditis with myocardial necrosis, degeneration, mineralization or fibrosis and hemorrhages are the main microscopic findings. Vasculitis can be seen in blue jays and American kestrels (*Falco sparverius*) [42,118] (Table 3). Cardiomyocytes, inflammatory and endothelial cells, and smooth muscle cells of arteries usually show immunostaining. Although the heart is usually affected in WNV infected birds, Anseriformes rarely have lesions in this tissue, even though viral antigen is demonstrated by IHC [71].

### 5.5. Spleen and other lymphoid organs

Splenic lesions can appear as early as 2 dpi in jays and crows, increasing in severity as infection progresses [64] but in falcons they may not be prominent until 7 dpi [72]. In this organ, lymphoid necrosis or apoptosis with fibrin deposition and hemosiderosis are consistently observed. Hemorrhages are described in ducks, guanay cormorants, and black-crowned night heron (*Nycticorax nycticorax*) [71] and lymphoid depletion in goshawks and owls [26,58] (Table 3). Viral antigen is detected in dendritic cells/macrophages and smooth muscular cells of arterioles. Necrosis can be found in bursal and thymic lymphoid cells [119,124] but these tissues have only been analyzed in some naturally WNV exposed birds, most probably because they are only present in juvenile birds (Table 3).

### 5.6. Liver

Similarly to the spleen, hepatic lesions can be found in crows as early as 3 dpi and increase in severity during progress of the infection [64]. In owls and falcons on day 5 pi these lesions are mild changing to severe by day 14 pi [66,72]. Lymphoplasmacytic and histiocytic hepatitis as well as coagulative hepatocyte necrosis are the most frequent lesions. Hemorrhages in goshawks and blue jays [26,118], biliary duct hyperplasia in northern bald eagles (*Haliaeetus leucocephalus alascanus*) and Chilean flamingos (*Phoenicopterus chilensis*) [71], and vasculitis in American kestrels can also be found [42] (Table 3). Birds that show hemosiderosis in the spleen usually also have it in the liver [119,120,122] (Table 3). WNV antigen is mainly seen in hepatocytes and Kupffer cells. Anseriformes do not show lesions [25] but WNV antigen is detected by IHC.

### 5.7. Kidney

Renal lesions can be moderate on 6 dpi in crows [62] and 9 dpi in owls [66] but may not become apparent until 14 dpi in falcons [72]. Lymphoplasmacytic and histiocytic interstitial nephritis and tubular epithelial cell degeneration and/or necrosis are detected in most WNV infected birds. Glomerular cell degeneration and/or necrosis are described in birds of the order Psittaciformes and Strigiformes [87,90] and so are hemorrhages in ducks [124] and vasculitis in American kestrels [66] (Table 3). IHC demonstrates WNV antigen in tubular and collecting duct epithelial cells, glomerular cells, infiltrating inflammatory cells, interstitial fibroblasts and, less frequently, in endothelial cells.

### 5.8. Lung

WNV antigen labeling and associated lesions are not usually described in the lung or if present they are mild. Lymphohistiocytic inflammatory infiltrates have occasionally been described and interstitial edema and necrosis can also be found. In naturally WNV infected Anseriformes and Psittaciformes pulmonary lesions have not been documented [123,124] (Table 3). Viral antigen is detected mainly in inflammatory cells although it can also be found in epithelial cells of the air capillaries.

### 5.9. Gastrointestinal tract

Lesions in the gastrointestinal tract are mainly described in the proventriculus, ventriculus and intestines with enterocyte and intestinal crypt cell necrosis, lymphoplasmacytic and histiocytic infiltrates, and proventricular gland and ventricular hemorrhages as main microscopic findings. In owls, but even less so in goshawks, lesions in the gastrointestinal tract are rarely found or if present changes are mild [26,82] (Table 3). Viral antigen is usually detected in enterocytes and intestinal crypt cells, inflammatory cells and smooth muscle cells of the lamina muscularis.

### 5.10. Endocrine system

Lymphoplasmacytic and histiocytic pancreatitis and acinar cell necrosis are present in most species (Table 3). WNV antigen is detected in exocrine cells. Adrenalitis has been described in different bird species but adrenal gland cell necrosis has only been documented in Psittaciformes [87] (Table 3). Viral antigen is present in both cortical and medullary cells. Thyroid gland has rarely been analyzed in the available studies but follicular cell necrosis has been described in ducks [124] and thyroiditis in goshawks [91] (Table 3). Viral antigen in follicular cells has only been described in owls [90] and goshawks [26,91].

### 5.11. Gonads

WNV associated lesions in gonads have only been documented in owls in which the authors described oophoritis, epididimitis and necrosis of granulosa cells, oocytes and seminiferous tubular cells [90] (Table 3). Viral antigen has been detected in different species in oocytes, theca and granulosa cells, stromal cells, seminiferous tubule cells and infiltrating inflammatory cells.

### 5.12. Skeletal muscle

In the skeletal muscle WNV infection can produce myositis, myofibril degeneration, necrosis and fibrosis (Table 3). IHC shows viral antigen in myofibers but this is not consistently observed [123].

### 5.13. Skin

Lymphocytic dermatitis has only been described in WNV infected Psittaciformes [87] (Table 3). Viral antigen is detected in keratinocytes of the basal layer of the epidermis, fibrocytes and macrophages of the dermis and subcutis, interstitial cells and epithelial cells of the feather pulp. Although in raptors and owls skin lesions are not described, viral antigen is usually detected [91].

### 5.14. Bone marrow

Cellular necrosis in American crows [80] and hypercellularity in turkeys [122] are the only lesions that have been described in the bone marrow (Table 3). WNV antigen can be detected in hematopoietic cells.

Despite all the cited differences among bird orders, families or even species, lesions and viral antigen are described in the majority of organs in WNV infected birds (Figure 1).

## 6. Discussion and conclusion

West Nile virus, a pathogen considered of little importance for birds before the 1990’s [47], is nowadays one of the most widely distributed arboviruses in the world that causes thousands of bird deaths, with a locally significant impact on populations of native species in North America [9,125].

In this work we have reviewed the pathogenesis of WNV infection in experimentally infected birds and the main pathologic changes that have been described in natural infections. The information that has been reviewed indicates on one hand that in most infected birds the virus first appears in the spleen after which it spreads rapidly to other organs such as the kidney, lung, heart and liver, reaching later the CNS. However, tissue distribution takes place earlier in bird species highly susceptible to the infection such as Passeriformes and later in less susceptible species such as Falconiformes, Galliformes or Strigiformes. On the other hand, the reviewed information indicates that most infected birds that show clinical disease and some of those that die without previous clinical manifestations have macroscopic and/or microscopic lesions, but that there may be differences in their distribution and severity between species even within the same family. Accordingly, considerable differences exist among species in expression of clinical disease that are not always clearly related to the severity and distribution of lesions. For example, nervous clinical signs do not always correlate with the pathological findings in the brain such as neuronal necrosis.

Acute lesions such as encephalitis and hemorrhages in different tissues are consistently found in the vast majority of the described bird families. Hemorrhagic fever associated to WNV infection has mainly been described in humans [126,127] in which hemorrhages are thought to be related to direct or indirect microvascular damage [127]. Thus similarly, the vasculitis and endothelial WNV antigen found in some bird species could explain the presence of blood extravasation. A study on human pathogenic flaviviruses has associated sequence signatures in the envelope protein of the virus with the primary syndrome that they produce (encephalitis or hemorrhagic disease) [128], but envelope protein sequence is not available for most WNV strains that infected the birds from which lesion descriptions exist. One lesion that is not consistently found in every bird family or even in different species within families is hemosiderosis in the spleen and liver. Avian hepatic hemosiderosis has been observed in relation to iron overload in captive wild forest birds but is also frequently associated to hemolytic processes in acute infectious disease [129]. Although Passeriformes develop necrosis and mild inflammation in the heart, spleen, liver or kidney, they show only mild encephalitic lesions and neuronal necrosis is absent [71,80,121]. This may be related to the higher susceptibility of this order to WNV infection leading to rapid viral distribution and host death that does not allow development of encephalic lesions [118]. The absence of encephalitis in juvenile chukar partridges (*Alectoris chukar*) and Impeyan pheasants (*Lophophorus impeyanus*) [83] could have the same explanation. In contrast, neuronal degeneration, necrosis and phagocytosis are well documented in Strigiformes and Falconiformes, potentially because the course of the disease in these species is more prolonged. The detection of WNV antigen or genome by IHC or real time RT-PCR in a particular tissue, even when there are no observable macroscopic or microscopic lesions, or the apparent absence of viral antigen immunolabeling although microscopic related lesions are present, may have the same underlying cause [90,118]. Thus, lesion description and viral antigen detection should be considered together in order to enable us to understand the pathogenesis of WNV infection in a particular host.

Many pathologic differences are related to the time post-infection the animal died which is related to its susceptibility to infection and immune capacity at the time the virus is inoculated, as well as the infection route (oral vs. mosquito), the infective dose and the virulence of the infecting virus. Looking for example at the description of Lopes et al. [79], species specific factors are evident as in this case the infecting virus, route and dose were probably very similar. Based upon the evaluated information, apparently there are species in which the individuals respond early and strongly to the infection clearing the virus rapidly, suffering thus only mild microscopic lesions and causing only few individuals to succumb to the disease [72]. On the contrary, other species potentially mount a weak immune response of late onset that leads to high tissue viral loads and death due to the severity of tissue lesions during the first general viremia [62,68]. Finally, there is a third group of species which maintains low levels of viral replication that leads to chronic infection which can finally produce host death or long-term sequelaes [42]. Nevertheless, it is important to consider that the literature about the pathology of natural WNV infection in birds is limited to a number of species that may not necessarily reflect the complete host spectrum of species affected by natural disease. Thus, the information is unbalanced, meaning that the absence of lesions in certain tissues in any particular bird species could be related to the limited number of studies available. The reason why most studies about the pathology of WNV infection have been carried out in raptors and owls may be related to the higher visibility of dead or clinically affected animals of these species in the field. Furthermore, these animals are frequently maintained in facilities such as zoos or rehabilitation centers where they are observed daily allowing to perform necropsies of fresh carcasses in case of death. Additionally, studies in corvids in the US are probably driven by the magnitude of mortalities in these species and their visibility for the public as they are relatively abundant in urban and suburban areas. The lack of information about WNV infection pathology in other species could be related to the lack of fresh carcasses that enable a detailed pathological description.

Certain bird species considered resistant to the disease in the field such as the red-legged partridge (*Alectoris rufa*) develop lesions and succumbed to the disease in experimental studies [84]. Therefore, it is important to consider that lesion and viral antigen distribution between experimentally and naturally infected bird species is probably different, and that numerous contributing factors in the field are generally not reproduced under experimental conditions.

In conclusion, differences in pathology of WNV infection in different bird species are most probably related to a combination of host and environmental factors in addition to the intrinsic virulence and pathogenicity of the infecting virus strain. The non-progressive, acute or more prolonged course of the disease accounts for part of the differences in the distribution of lesions and viral antigen and in the severity of lesions. Finally, although experimental infections cannot completely reproduce field situations, they can increase our understanding of pathologic pathways and help in the identification of key factors that influence the outcome of an infection in a given host.

## 7. Competing interests

The authors declare that they have no competing interests.

## 8. Authors’ contributions

UH proposed the subject, wrote the outlines and reviewed and edited the paper, VG compiled the information, wrote and edited the paper. Both authors agreed on the final version of the manuscript.

## 9. Authors’ information

VG is a grant student in avian pathology at IREC and has a special interest in flavivirus infections. Her supervisor UH investigates and teaches on infectious diseases in wild birds.
